# Building a strategic educator–psychiatrist alliance to support the mental health of students during the outbreak of COVID-19 in China

**DOI:** 10.1017/gmh.2020.27

**Published:** 2020-11-10

**Authors:** Ding Ren, Lixia Wang, Xiao Pan, Yonghai Bai, Zhengmei Xu

**Affiliations:** 1 Department of Medical Psychology, PLA Navy No. 905 Hospital, Second Military Medical University, Shanghai, 200052, P.R. China; 2 Shanghai Teacher Training Center, Shanghai, 200234, P.R. China; 3 Department of Medical Psychology, Shanghai Changzheng Hospital, Second Military Medical University, Shanghai, 200003, P.R. China; 4 Shanghai Changzheng Hospital, Second Military Medical University, Shanghai, 200003, P.R. China

COVID-19, initially identified in Wuhan, Hubei Province, has widely and rapidly spread across China and in many other countries around the world, causing an outbreak of acute infectious pneumonia. The unpredictable future of this epidemic has been exacerbated by myths and misinformation, often driven by erroneous news reports and the public's misunderstanding of health messages, thus causing anxiety in the public. It has been reported that the outbreak of COVID-19 in China has caused public panic and mental health stress (Chen *et al*., 2020; Duan and Zhu, 2020; Liem *et al*., 2020; Liu *et al*., 2020; Yang *et al*., 2020). Studies have also confirmed that some individuals who have experienced public health emergencies may still have varying degrees of stress disorders, even after the event is over, or after they have been cured and discharged from hospital, indicating that these individuals may need extra care and should not be ignored (Fan *et al*., 2015).

Since the outbreak of COVID-19, schools have been closed in China. The psychological impact of school closure on students could be significant because they are isolated from the normal school environment. As we know, the school environment not only refers to the physical space, but also includes the emotional and cultural space (Mura *et al*., 2015). Positive relationship experiences in school, such as peer belonging and teacher support, are significant contributors to students' positive mental health (Oberle *et al*., 2018). As students are required to study at home, the campus cultural space, the mutual companionship between classmates and the emotional exchange between teachers and students no longer exist. Students are likely to become nervous, anxious, and even panic-stricken (Main *et al*., 2011). The psychological impact of quarantine may be similar to the effects of sensory deprivation which include confusion, boredom, irritability, and difficulty in concentrating. Indeed, a review on psychological impact of quarantine found that various symptoms such as emotional disturbance, depression, stress, low mood, irritability, insomnia, post-traumatic stress symptoms, anger, and emotional exhaustion had been reported (Brooks *et al*., 2020). Therefore, it is urgent to assist students to adjust their psychological state and stabilize their mood. Home confinement may also affect students' physical health because they are physically less active and may have irregular sleep patterns and less favorable diets which will result in weight gain and a loss of cardiorespiratory fitness (Wang *et al*., 2020). Wang and his colleagues analyzed the effects of home confinement on children’ physical and mental health during the COVID-19 outbreak, and suggested that the government, non-governmental organizations, the community, school, and caregivers should take timely and sufficient actions to tackle these issues in China (Wang *et al*., 2020).

**Fig. 1.**
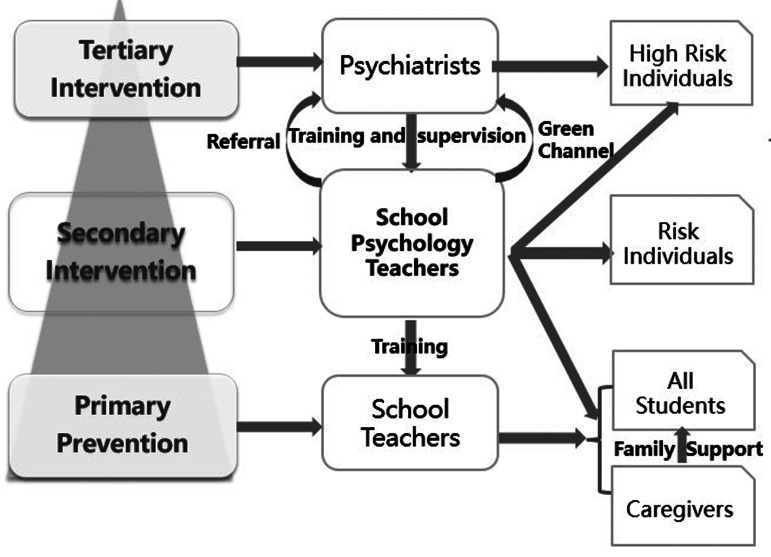
Three-tiered psychological support system for students and staff.

The education authorities at all levels responded promptly to the outbreak of COVID-19. Chinese educators are fully aware that the educational function of schools under normal circumstances is largely realized through the school environment, and that the school space is an environment specially created on the basis of the psychological and moral tendencies that affect its members (Mura *et al*., 2015). They started to provide mental health support at the beginning of the epidemic outbreak, implemented *Guidelines of Emergency Psychological Crisis Intervention for COVID-19 Infection* and *Guidelines for Psychological Assistance Hotline for the Prevention and Control of COVID-19* issued by the National Health Commission (2020), and provided services including psychological support hotlines and online counseling. Among these effective actions, the most valuable and innovative support for students is the establishment of the Education System Epidemic Prevention Alliance, a joint effort between Chinese educators and mental health professionals. This alliance is mainly aimed at elementary school students, junior high school students, and high school students. In Shanghai, the schools that were involved launched quick and effective actions to ensure the mental health of teachers, students and caregivers after the COVID-19 outbreak in November 2019, based on a three-tiered psychological support system (TTPSS) (Fig. 1) constructed by the Shanghai Teacher Training Center and the Medical Psychology Department at Shanghai Changzheng Hospital.

The TTPSS mainly addresses mental health issues of students and the professional competency enhancement of school psychology teachers who are responsible for delivering psychology courses and offering psychological counseling at school. The system is based on the Biopsychosocial Model, which provides the basic paradigm for mental health as it is now broadly accepted that illness and health are the result of the interaction of biological, psychological, and social factors (Engel, 1980; Read *et al*., 2008; Alvarez *et al*., 2012), and derives from the Tertiary Disease Prevention Model, which includes three categories of prevention, aiming to prevent disease or injury, reduce the impact of disease or injury, and soften the impact of an ongoing illness or injury respectively (Institute for Work & Health, 2015).

At the primary level, all school teachers are trained by school psychology teachers to obtain the general knowledge of psychology in order to popularize it and education students and caregivers about it. The goal at the primary level is to prevent emotional and behavioral disorders such as anxiety and depression. School teachers give lessons to students and caregivers. The lessons targeted at students include emotional control, psychological resilience improvement, and learning-related anxiety management. The lessons for caregivers include caregiver–child communication and stress management. Thus, the caregivers can provide more beneficial family support to students. The interaction between students through face-to-face communication or social network apps such as DingTalk, WeChat, and QQ will also construct favorable peer support relationships.

At the secondary level, school psychology teachers are responsible for establishing mental health profiles of students, screening students for potential mental health problems, and identifying at-risk individuals for early intervention. Psychology teachers are recommended to attend training courses organized by the Shanghai Teacher Training Center, where they are trained and supervised by psychiatrists from the Medical Psychology Department of Shanghai Changzheng Hospital. The training includes Diagnostic and Statistical Manual of Mental Disorders (DSM-5), solution-focused brief therapy, family therapy and mindfulness, etc. If school teachers notice that a student shows some symptoms of psychological disorders such as anxiety or depression, they will refer the student to school psychology teachers who will assess his/her psychological level through interviews and assessment scales such as the Psychological Symptom Checklist (SCL-90). Based on interviews and case conceptualization, and also taking into account the results of the scales, school psychology teachers will offer intervention to students at risk of psychological trouble through a variety of approaches, including cognitive behavior therapy (CBT), mindfulness, social emotional learning (SEL), etc. CBT is used to alleviate anxiety, mindfulness for relaxation or self-regulation, and SEL to teach communication skills (Arora *et al*., 2019; Kirk *et al*., 2019). CBT and mindfulness training are conducted both individually and in groups, whereas SEL is mostly conducted in groups.

At the tertiary level, school psychology teachers refer high risk individuals to psychiatrists. Psychiatrists from the Medical Psychology Department of Shanghai Changzheng Hospital offer medical treatment and psychological therapy. School psychology teachers also offer psychological interventions in collaboration with psychiatrists and students' families.

During the outbreak of COVID-19, trained school psychology teachers worked quickly with psychiatrists to initiate effective actions. They carried out online mental health services which focused on at-risk individuals. They used a number of scales developed for measuring the impact of COVID-19 on mental health, for instance, the Fear of COVID-19 Scale (FCV-19S) (Ahorsu *et al*., 2020) and Coronavirus Anxiety Scale (CAS) (Lee, 2020), as screening scales. They developed a series of online Micro-Sessions on psychological wellbeing, and established a green channel for students with severe mental disorders to seek medical treatment. Trained school teachers popularized psychological and epidemic knowledge among students and caregivers to decrease public panic and mental health stress during the COVID-19 epidemic. These actions enabled teachers to help maintain and develop students' mental health in a timely manner.

However, in face of the high demand for mental health care among students, caregivers, and teachers following the COVID-19 outbreak, the supply of professional psychological support in schools in China is insufficient. It is important to train a pool of qualified professional school psychology teachers through preservice teacher education. School psychology teachers need further training and mentoring in clinical psychology to quickly identify at-risk individuals in schools. Therefore, there is an urgent need for China to build a mental health promotion alliance mainly composed of educators and psychiatrists. We hope that education authorities would pay special attention to the development of applied clinical and counseling psychology disciplines and make it imperative to build effective referral channels in schools for medical interventions. Moreover, while carrying out the TTPSS, there is a need for systematic study of the implementation process of the mental health services on multiple levels: student level (e.g. mental health outcomes, satisfaction with services, etc.); parent outcomes (e.g. attitudes toward the services and changes in their children); teacher and school administration outcomes, etc. Such studies would inform both researchers and practitioners of potential issues in the implementation of the TTPSS with an eye to ensure its effectiveness.
